# Enhancing Diagnostic Precision: A Distribution-Based Compressed Denoising Scheme using Transfer Learning for Noise Reduction in Medical Imaging

**DOI:** 10.2174/0115734056391923250827170359

**Published:** 2025-09-17

**Authors:** Shtwai Alsubai, Waseem Ahmad, Mohd Anjum, Ashit Kumar Dutta, Sana Shahab, Assefa Senbato Genale

**Affiliations:** 1 Department of Computer Science, College of Computer Engineering and Sciences in Al-Kharj, Prince Sattam bin Abdulaziz University, Al-Kharj 11942, Saudi Arabia; 2 Department of Computer Science and Engineering, Vishveshwarya Group of Institutions (VGI), Gautam Buddha Nagar 201314, Uttar Pradesh, India; 3 Department of Computer Engineering, Aligarh Muslim University, Aligarh 202002, India; 4 Department of Computer Science and Information Systems, College of Applied Sciences, AlMaarefa University, Ad Diriyah, Riyadh 13713, Kingdom of Saudi Arabia; 5 Department of Business Administration, College of Business Administration, Princess Nourah Bint Abdulrahman University, Riyadh 11671, Saudi Arabia; 6 Department of Computer Science, College of Computing and Informatics, Bule Hora University, Bule Hora, Ethiopia

**Keywords:** Medical imaging technology, Transfer learning, Compressed denoising scheme, Healthcare diagnosis precision, Noise reduction

## Abstract

**Introduction:**

Accurate interpretation of medical images is vital for diagnosis and treatment planning. However, noise in medical imaging significantly hampers this process, leading to potential diagnostic errors. Traditional denoising techniques often require large datasets or compromise diagnostic details.

**Method:**

This study proposes a Distribution-based Compressed Denoising Scheme (DCDS) leveraging transfer learning to enhance diagnostic precision. The framework analyzes pixel distributions to distinguish normal and noisy pixels using incremental and decremental distribution verification. A chest CT scan dataset with Gaussian-like noise was used to train the model, employing correlation mapping between training and target images for effective variance estimation.

**Result:**

Experimental analysis showed that DCDS significantly improves noise reduction, variance detection, and diagnostic precision. The peak signal-to-noise ratio improved across all evaluated ranges (24–32 dB), with a noise reduction rate exceeding 82% in optimal conditions. DCDS reduced mean error and analysis time, enhancing overall system efficiency. Transfer learning allowed for effective pixel classification and noise prediction with fewer data and computational resources.

**Discussion:**

Compared to existing denoising methods, DCDS offers better generalization through statistical modeling and knowledge-sharing between training and learning states. However, current validation is limited to CT imaging with Gaussian noise. Broader modality testing and integration of perceptual and clinical grading metrics are needed.

**Conclusion:**

The DCDS framework presents a promising, lightweight, adaptive, and accurate solution for medical image denoising. It holds potential for real-time diagnostic support, but further optimization and clinical validation are necessary to ensure generalizability across diverse imaging modalities and noise types.

## 1 INTRODUCTION

### 1.1 Background and Importance

Compressed denoising is an effective method for removing noise in images by compressing the data before denoising it. To get rid of extra noise and enhance the quality of healthcare pictures, diagnostic denoising is important [1]. We can improve healthcare imaging by using compressed denoising techniques, including Wavelet Transforms, Gaussian Filters, Median Filters, and Autoencoders, as well as more sophisticated approaches, such as Convolutional Neural Networks (CNNs) and compressed sensing [2]. Deep learning (DL) algorithms, especially autoencoders and CNNs, are among the hardest ones to use [3]. Neural networks that have been trained on a large amount of data may find complicated patterns and links in medical pictures, even when they are poorly lit [4]. It is very important to remove noise, especially in these challenging circumstances. When choosing denoising methods, factors including the type of noise, the computer’s processing speed, and the type of imaging are all taken into account [5]. Healthcare picture denoising with high diagnostic precision helps doctors to make better sense of medical images [6]. Compression methods are crucial for keeping important diagnostic data safe, as they reduce the size of the picture file [7].

The Discrete Wavelet Transform can assist with lower compression noise [8]. Lossless approaches, such as Huffman coding, are particularly helpful for denoising since they preserve the details of the picture without dramatically compromising the quality [9]. JPEG is a lossy compression method that can aid in denoising, although better quality settings can still help preserve important clinical data [10]. Run-length Encoding is more effective than many other compression methods, as it has a significant effect on reducing noise [11]. When choosing compression methods, it is important to strike a balance between retaining diagnostic data and minimizing data loss as much as possible [12]. Researchers are working hard to improve blurring and compression methods to make healthcare picture storage and transmission more efficient [13].

To achieve the best results in medical imaging denoising, both old and new methods are typically used together. Gaussian filters and Wavelet transformations are two examples of traditional mathematical models that are often employed to get rid of noise [14]. Modern neural networks use powerful models, such as autoencoders and CNNs, to identify patterns and complex relationships in photos taken under various lighting situations [15-17]. To assess how well the system works, you need to evaluate how well it can decrease noise while preserving important medical data. This method makes healthcare diagnostic systems more accurate [18].

### 1.2 Related Works

He *et al*. developed an Attentive Generative Adversarial Network to eliminate noise in Photoacoustic Microscopy (PAM) pictures [19]. Their objective is to continually improve the quality of PAM images so that they can be used more often in medicine. The proposed method uses an attention-enhanced Generative Adversarial Network with strong DL capabilities to make PAM pictures less noisy in real-time.

Lee *et al*. developed in with an Interdependent Self-Cooperative Learning method for removing noise from clinical photos that were not matched [20]. Their research shows that this method might help make medical picture denoising better. The proposed technique employs cyclical antagonistic learning and self-supervised residual learning to accomplish effective noise reduction.

Niu *et al*. did a thorough analysis of Noise2Sim with the goal of reducing noise in medical imaging, especially in low-dose simultaneous photon-counting Computed Tomography (CT) [21]. The goal of this method is to make diagnoses more accurate while reducing radiation exposure.

Zhang *et al*. developed a new way to remove noise from Low-Dose CT (LDCT) images by combining transformers with CNNs [22]. The study's goal was to improve the visual quality of LDCT pictures, which would make diagnoses more accurate.

To improve the quality of CT pictures, Hou *et al*. built a dual-channel artificial intelligence neural network system [23]. When applied to high-resolution images, the denoising technique not only enhances the quality of CT images but also lowers the amount of X-ray energy required.

Khowaja *et al*. developed a flexible and useful method for removing noise from photos called Cascaded and Recursive ConvNets [24]. The main goal of this work was to find solutions to difficulties with inverted photography that typical discriminative learning approaches can not handle well.

Uetani *et al*. performed a comparative study to improve pituitary Magnetic Resonance Imaging (MRI) by comparing old methods with a new computer-based method [25]. The researchers looked at how well combining wavelet transforms with DL reconstruction worked for undersampled hypothalamic MRI signals.

The non-local Linear Minimum Mean Square Error technique was proposed by Rahimizadeh *et al*. as a new way to reduce speckle noise in ultrasound images [26]. The study's goal was to significantly improve the performance of the suggested approach by utilizing the built-in redundancy in ultrasound pictures.

Prigent *et al*. developed a highly flexible method to quickly deconvolve and denoise 3D fluorescence microscope images and films [27]. This method solves problems with optical distortions and limited photon budgets that are common in current fluorescence microscopy.

In a study by Xu *et al*., the authors improved the Deep Image Prior network for removing noise from images by adding a three-stage approach that uses both generative and fusion algorithms [28]. They used internal and external prior-based denoisers to make two initial denoised pictures. Then, they used a spatially random mixer to make a variety of target images. The quality of the images was better because a weight map from an unsupervised generative network creates and combines many complementary samples.

Based on the U-Net architecture and a multi-attention technique, Zhang *et al*. proposed a new way to remove noise from CT images [29]. The main use of this method was to get rid of noise from low-dose medical CT scans. The technology uses the U-Net architecture and a multi-attention mechanism to eliminate noise while preserving important clinical information in CT images.

Ahmed *et al*. developed a method to remove noise from medical images using a stacked convolutional autoencoder [30]. The main purpose of the work was to simplify the removal of noise in two-dimensional electrophoresis gel pictures used in medical applications.

Wang *et al*. developed a method to get rid of noise in LDCT images utilizing self-supervised guided distillation of information [31]. The main goal of this procedure was to eliminate noise and aberrations in LDCT images. The method significantly increases the quality of LDCT pictures by using both knowledge distillation and self-directed learning.

Deng *et al*. developed a way to get rid of noise in gamma-ray photographs by dividing speckles [32]. Complementary Metal-Oxide Semiconductor detectors commonly produce images with significant noise in radiation conditions, and this technology tries to fix that problem.

Göreke *et al*. came up with a method to reduce Poisson noise in medical X-ray pictures using the Wiener filter [33]. The main purpose of this method was to improve the filter's performance by fixing problems like excessive picture smoothing, texture distortion, lower quality, and high computational needs.

Dhote *et al*. developed a Distributed Analytics of Data and Organisation Model to make it easier to handle medical data using mobile apps [34]. This method makes services more reliable by using dispersed cloud services to reduce problems with data storage and reorganization.

Even with these improvements, an enormous gap remains in the literature: there is no effective, generalizable, and data-efficient denoising algorithm that keeps clinical images intact across various imaging environments. Current solutions either eliminate structural information to reduce noise or require large datasets and substantial computing power. These are not practicable in many real-world healthcare situations, especially when resources are constrained. These clinical and computational demands that remain to be satisfied make this effort necessary. When noise removal is done wrong, it has a direct impact on diagnostic results. Additionally, Artificial Intelligence (AI)-based technologies cannot be employed in real-time clinical workflows since there are no adaptive, lightweight denoising solutions. There is an urgent need for an approach that improves image quality while also operating well with current diagnostic workflows.

### 1.3 Problem Statement

DL and wavelet-based algorithms are just two examples of the many techniques used in the medical image denoising field. While progress has been made in reducing noise and enhancing image quality across various modalities, several research gaps remain. These gaps include topics such as improving denoising through technique integration, thoroughly validating DL algorithms, ensuring interpretability, developing resource-efficient solutions for real-world healthcare, and testing resilience across different datasets and clinical scenarios. Addressing these challenges in denoising approaches can significantly enhance the accuracy of diagnoses and patient care, making the techniques more effective, interpretable, and practically applicable in clinical settings.

Noise poses a significant challenge in medical imaging, hindering the accurate detection and treatment of diseases. If left unaddressed, noise can lead to inaccuracies in diagnosis and misinterpretations, even with conventional denoising approaches. The need for advanced denoising methods arises from the limitations of existing techniques, which often struggle to balance noise reduction and the preservation of critical diagnostic data. This study proposes a Distribution-based Compressed Denoising Scheme (DCDS), a transfer learning-based system designed to enhance medical diagnostic accuracy.

### 1.4 Research Gap

There is an enormous gap in the present research, as there are no adaptable and resilient denoising frameworks that can operate with diverse types of noise without losing image quality. DL-based algorithms are more effective, but they require a substantial amount of high-quality, annotated data to work. This makes these individuals less effective in real-world healthcare settings, where such data is often hard to find or expensive to acquire. Only a few studies have used transfer learning to modify pre-trained models for denoising tasks in a compressed representation framework. This can lower the amount of computational resources required and enhance generalization

### 1.5 Contributions

The key contributions of this study include:

(a) Proposing a DCDS to enhance healthcare diagnostic precision.

(b) Establishing a correlation between pixel distribution and variation within training data to differentiate normal and noisy pixels.

(c) Introducing a gradual and decremental dispersion verification technique that facilitates collaboration between training and learning states through knowledge sharing.

(d) Conducting experimental analysis using real-time medical images for noise suppression.

(e) Performing comparative analysis across various metrics, including Peak Signal-to-Noise Ratio (PSNR) variations.

PSNR is a widely accepted quantitative measure of signal fidelity; nonetheless, it largely represents accuracy at the pixel level and does not always align with human visual perception or clinical diagnostic value. A number of denoising techniques, in fact, are capable of achieving high PSNR values, but at the expense of introducing artifacts or blurring that diminishes visual clarity and hides essential anatomical details. We are aware that the aforementioned limitation affects the comprehensiveness of our performance evaluation procedures. In order to solve this issue, future research will use other perceptual quality metrics, such as the structural similarity index measure, which takes into account brightness, contrast, and structural fidelity in order to provide a more perceptually aligned evaluation. Visual information fidelity and feature similarity index are two examples of sophisticated visual quality indicators that we intend to incorporate into our system. Additionally, we intend to incorporate expert-based visual grading by radiologists in order to assess clinical readability. Furthermore, we intend to evaluate the effect that denoising has on subsequent diagnostic tasks, with the goal of ensuring that improvements in picture quality offer a meaningful contribution to the process of clinical decision-making. These modifications will result in an evaluation methodology for the proposed DCDS model that is more robust and therapeutically appropriate with regard to its evaluation.

This study presents a DCDS aimed at enhancing healthcare diagnostic accuracy. By analyzing the pixel distribution in comparison with training data, the proposed approach gradually verifies dispersion and effectively differentiates between normal and noisy pixels. The performance of the proposed denoising scheme is evaluated through noise suppression analysis using various metrics and modifications in PSNR ranges, tested on real-time medical images. Table **1** provides a list of notations used in this study.

## 2 MATERIALS AND METHODS

This proposal focuses on improving diagnostic precision in medical image denoising by utilizing DCDS, which integrates transfer learning to identify noisy and normal pixels. The pixel identification process is based on image detailing and smoothing, which aids in the diagnostic interpretation of medical images. In the DCDS framework, transfer learning is applied to predict pixel distributions by processing training images, allowing for the identification of noisy and denoised pixels. The medical image, serving as health-related input, undergoes analysis to detect and denoise, with the final prediction facilitated by transfer learning. A schematic illustration of the DCDS scheme is shown in Fig. (**1**).

In this work, the training set is derived from the existing procedure that considers pixel distribution. Regions are extracted from the input medical image (*e.g*., MRI, X-ray, CT), and smoothing is applied to enhance the diagnostic accuracy. The primary objective of this paper is to classify medical images with minimal noise, ensuring improved precision in medical image processing. The initial step involves analyzing the medical image through region-based extraction, which is then evaluated for its effectiveness in denoising and enhancing image quality. According to Eq. (**1**), the medical image analysis process is represented as *A_n,_* while the input image is denoted as *M_g_.* Additionally, region-based extraction is also performed in Eq. (**1**), which is described by *r’* and *e_x._* The image smoothing and detailing are denoted by *m_s_* and *lg*, respectively.

**Table:** 

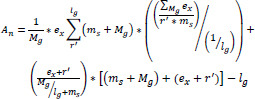	(1)

Through the above extraction process, the region of interest is identified, and the pixel distribution from the input image is determined. The initial step involves detecting the region based on image detailing, which indicates smoothing and diagnostic accuracy. The image detailing, which measures the number of medical images from which extraction is performed, is captured in the expression.


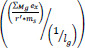


This approach focuses on image extraction, smoothing, and detailing to detect noisy pixels, which are crucial for the diagnostic process. The knowledge-based analysis used in this stage is based on region-based extraction. Subsequently, the categorization of pixel distribution and variance is calculated and equated in Eq. (**2**).

**Table:** 

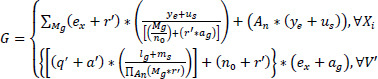	(2)

The categorization of pixel distribution and variance, denoted as *G*, is formulated with the pixel distribution *X_i_* and variance *V*’ in this context. Both the density *y_e_* and resolution *u_s_* are defined, influencing the image capture process. The noise is represented as *n_0_*, and both density and resolution are essential in ensuring the image’s diagnostic accuracy, denoted as *a_g_*. The pixel distribution is determined based on the input image, where extraction follows the region identification process, represented by the equation.


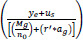


Variance, on the other hand, refers to how pixels are separated based on quality and value, denoted as *q*’ and *a*’. The extracted distribution and variance from the input image are validated and illustrated in Fig. (**2**).

**Fig. (2):**
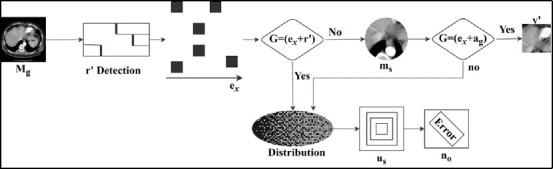
Distribution and variance estimation.

*M_g_* is the input used to categorize *V’* and *X’* from *r*’ available. In the categorization process *e_x_* variants are used to identify *m_s_* or *X’* in the initial phase. *G* = (*e_x_* + *r*’) is *X*’ the extraction condition for validating the distribution. The failing condition requires smoothing and detailing labels to identify *V*’. Therefore, if *G* = (*ex* + *a_g_*), then *n_o_* is present as variance, identified (Fig. **2**). Followed by the above illustration, *X_i_* and *V*’ for different *G* is tabulated in Table **1**.

In Table **1**, *X_i_* and *V*' for different *G* conditions are tabulated. (1/kg) and (*M_g_*/*n*_0_) are the classification conditions for each *r*’. This process is validated between *X_i_* and *V*’ for multiple *a_g_* and *y_e_*. In this early estimation, the correlation is based on both factors independently for *A_n_*. However, these features are revisited after *b_i_* and *σ* for multiple regions. This noise detection is established for region-based segmentation, where the pixel distribution is performed on the input image. Based on the pixel, the variance is used to calculate the possible identification, whether large or small. Here, the detailing is used for the image pixel distribution for the variance accomplishment, and it is.


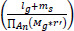


Hence, the categorization of the medical image relies on the pixel distribution and the variances, where the correlation factor is introduced to find the value among the pixels and the variance, which is processed from the training image and formulated in Eq. (**3**).

**Table:** 

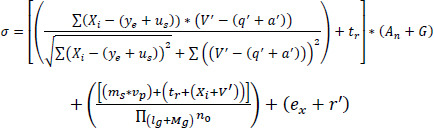	(3)

The correlation factor is used in this instance, and it is *σ*. This observation is performed for the pixel distribution and the variance, where the training image is used in this process, and it is described as *t_r_*. The convergence to optimal denoising is *v_p_*, which is used for solving the high range of denoising in the image. The density of the image is used to facilitate region-based extraction, where the analysis is followed up on for the input image and the noise detection process. The detection of noise is used to define the medical image, where the pixel resolution issues are detected. The density and the resolution are addressed in this pixel distribution, where it deploys the training image. The training image is trained with the predefined knowledge associated with the current approach.

The training image is accompanied by the variance, which is the ideal way to separate the pixel from the noise. The noise is detected by using these two methods. The training is built for further denoising detection, and it is formulated as.


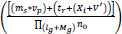


The extraction of the acquired region from the input image is used to provide better density, resolution, quality, and value-based computation. From this computation method, the correlation factor is formulated for reliable image processing in this work. By observing this, the distribution of incremental and decremental is represented in Eq. (**4**).

**Table:** 

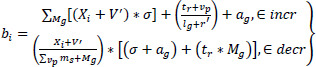	(4)

The distribution *b_i_* is performed for the incremental and decremental, and it is represented *incr* and *decr* as in Eq. (**4**). Here, it states, the training of the data in which the diagnostic is detected for the convergence to optimal denoising, and it is formulated as.


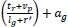


By incrementing the distribution process, the compressed image is used to detect noise better. In this category, the diagnostic is involved in the region-based extraction of the medical image. The analysis is carried out in this section for the incremental method, in which the correlation factor is involved in the betterment process. The increment and decrement categorization using *σ* is analyzed in Fig. (**3**).

**Fig. (3):**
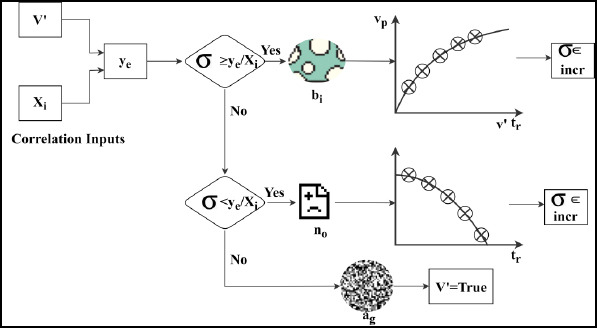
Increment and decrement categorization using *σ*.

*V’* and *X_i_* inputs are used for *y_e_* assessment for *σ*; the *σ* 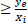 defines the incremental case. In this process, *b_i_* is validated as *v_p_* that does not require a pre-training model. Therefore, the number of training images is verified for *σ* under low *n_o._* There are two differential cases in this process: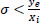 generates *n_o_* (new in *r*’) for which *t_r_* and *P_n_* are jointly required. This forms the decremental *b_i_* for any *e_x_*; the final differentiation is *V*’ = *True* where *X_i_ < V*’ is the case that identifies a high *n_o_*. Thus, the image generates less diagnostic support (Fig. **3**). The decremental method indicates the convergence to optimal denoising for the n-number of images. The computation stage is identified in this decremental distribution and enhances the process. For this, the training image set is used along with the correlation factor, and it is equated as [(*σ + a_g_*) + (*t_r_* * *M_g_*)]. From this stage, the incremental and decremental processes are used to enhance the diagnosis of medical images. The correlation follows incremental and decremented distribution verification with mutual knowledge (noise) transfer between the training and learning states. *b_i_* categorization for different regions under various conditions is tabulated in Table **2**.

*b_i_* value and category presented in Table **2** considers (*X_i_* + *V*’) and 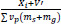 possibilities. In this case *e_x_* is the deciding factor for *n_o_* replacement/ resolution under different *r’*. *p_n_* is required for *σ* satisfying 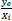 or *a_g_* factor. If the possibilities provide a maximum satisfying output (*V*’ + *X_i_*) = *true,* then *σ* is high; *b_i_* is identified for 3 possibilities that change in consecutive instances for *V*’ = *true* and *t_r_* (*decr*) cases. Therefore, final type categorization is defined based on max/min *σ.* The following section studies the transfer learning for this DCDS concept.

### 2.1 Transfer Learning for Classification

The initial viewpoint of this learning is proposed by training one image, which is used as the training model for the second image. The preceding information on the image is useful for learning about the upcoming image processing in this system. Transfer learning includes learning the features of the medical image from which the correlation factors are framed to process accordingly. The major assessment is to increase the prediction by gaining information from the previous set of images, which is a technique known as transfer learning. These steps are executed for the pixel distribution based on the knowledge of the input image. The pre-trained model is formulated in Eq. (**5**).

**Table:** 

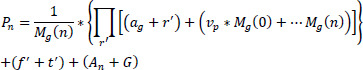	(5)

The pre-trained model given in Eq. (**5**) indicates the trained dataset, and it is described as *P_n_*. In this equation, the initial image is *M*_g_(0), the n-number of images is *M*_g_(*n*). Frequently, images are trained on the extension of the large dataset for future computation. The pre-trained model includes the features and patterns of the medical image into consideration and results in further task completion, and they are specified as *f and t’*. From this state of the pre-trained model, the identification of features and patterns is used for the optimal convergence denoising for the n-number of images, and it is represented as 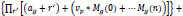

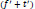
.

The state-of-the-art discussed in this pre-training model involves the analysis scheme for the development of the processing. The trained model is stored frequently and addresses issues such as density, resolution, quality, and value of the medical image. Based on these constraints, the pre-trained model gains the knowledge accordingly. From this approach, the pixel distribution and variance are associated with the correlation factor, where the incremental and decremented distributions are verified with mutual knowledge (noise). Following this pre-trained model, the base model is proposed in Eq. (**6**).

**Table:** 

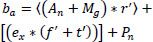	(6)

The base model includes the knowledge process, where the image is pre-trained along with the features and patterns, and it is represented as *b_a_*. The abstraction of the pre-trained model indicates the analysis of the image, where the extraction of the necessary features and patterns is associated with the training class. The base model is a hierarchical feature that is performed on the layers and provides the utilization of incoming data. The learning model for classification is illustrated in Fig. (**4**).

**Fig. (4):**
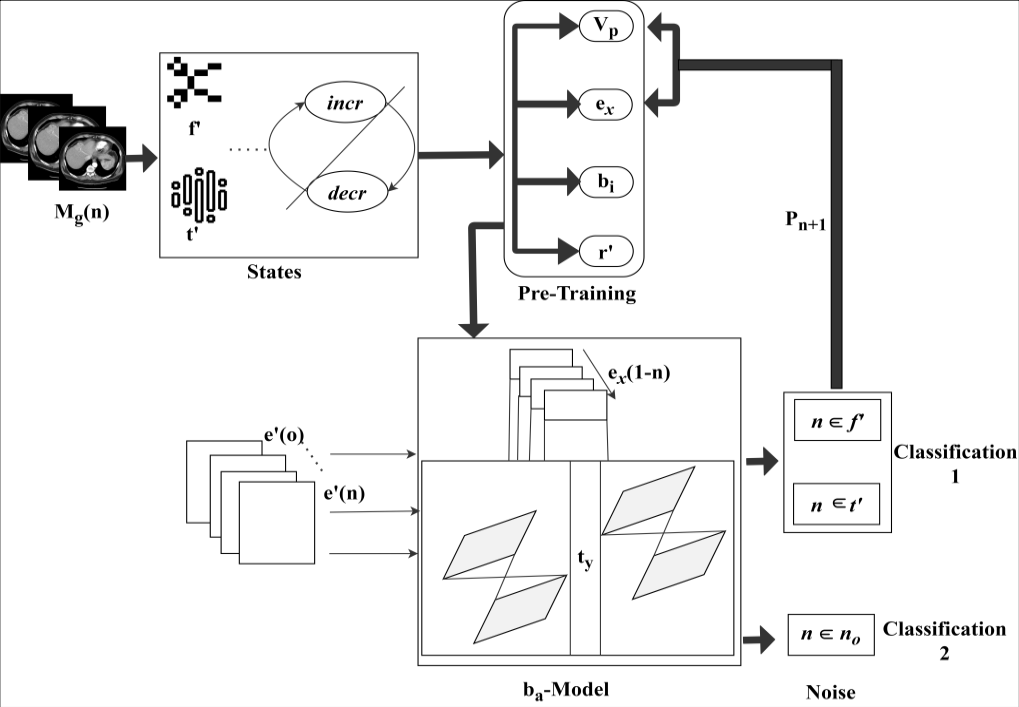
Transfer learning model for classification.

The learning for classification is reliable using *incr* and *decr* states of *P_n_* model. This pre-training model is responsible for pre-classifying (*v_p_* - *e_x_*) 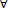 *incr* and (*bi*, *r*’) 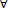 *decr*. Based on *b_a_* implication with *n* * *t_y_* layers *e*’(*o*) to *e*’(*n*) is mapped to validate if *σ* is high or *V’* is high. Considering the state changes from *P_n_* to *P_n+_*_1_, the classification knowledge is updated. The knowledge on *n_o_* and *n_o_* fewer pixel distributions between successive *P_n_* and *P_n+_*_1_ is required to classify (1 and 2) *r*’ (Fig. **4**). From this case, the feature and patterns are used to deploy a pre-trained model, and it is represented as [(e_x__*_ (*f*’ + *t*’))] + *P_n_.* Since the knowledge is shared in the image-based processing. The transfer layers are represented in Eq. (**7**).

**Table:** 

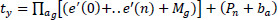	(7)

The transfer layer is responsible for forwarding the knowledge to the next layer in the network, and it is given in Eq. (**7**) as *t_y_*. Knowledge forwarding refers to the pre-trained model in this transfer learning. The obtained image is processed in the first layer, and it is *e’*(0), and the n-number of layer is *e’*(*n*). The information is based on the relevant task processing with the existing model detected in this transfer layer. This step of layer computation is associated with the pre-trained model and efficiently captures information regarding the image. From these transfer layers, the fine-tuning is detected in the section according to Eq. (**8**).

**Table:** 

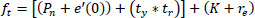	(8)

From Eq. (**8**), fine-tuning is described as the pre-trained dataset used to retrain the particular image in a certain layer, and it is labeled as *f_t_.* In this format, the re-training distinguishes the pre-training model and provides the knowledge for the selected layer. The knowledge is represented as *K*, and the re-training is described as *r_e_*. The accurate layers are responsible for forwarding the relevant information along with the specific knowledge gained from this fine-tuning. *r_e_* for different pre-training values are tabulated in Table **3**.

In Table **3**, *r_e_* demands from *incr* and *decr* states of the transfer learning module are presented. The considerations are *e_x_*, *v_p_, b_i_* and *r*’ for which if the operation is true, then it is 1 else 0. *ft* differentiations for high PSNR under compressed *n_o_* retards within *P_n_*. Depending on *t_y_* required for *P_n_* to *P_n+_*_1_ transition, the classification is required for *n* ϵ *f’* and *n* ϵ *t’* (classification 1 in Fig. **4**). If the states fail to be classified under either of the above cases, then *n* ϵ *n_o_* classification requires a new *P_n+_*_1_ where *r*_e_ is the initializing factor. This is further computed from *f_t_* in any *e*’(*n*) layer for classification. Based on this abstraction of knowledge sharing, retraining a particular layer from the dataset is said to be fine-tuning. All four models are used in this study to enhance transfer learning. Based on these models, the classification is derived for detecting the variance range, whether high or not, which examines the pre-train model and matches the noise pixel formulated in Eq. (**9**).

**Table:** 

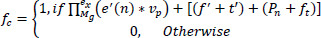	(9)

The classification is processed where variance is high or low and is distinguished as if and otherwise (given as Eq. (**9**). The classification is represented as *f_c_*, in which the pre-trained model indicates the fine-tuning model in the transfer learning. This condition is satisfied by using the transfer learning model that involves fine-tuning a pre-trained model. Here, both the feature and patterns are associated with the pre-trained and fine-tuning method, and it is represented as [(*f*’ + *t*’) + (*P_n_* + *f_t_*)]. If the condition is satisfied, the noise pixel is identified with a high variance. On the other hand, the other condition works if no noise is identified. The denoising of the medical image is represented in Eq. (**10**).

**Table:** 

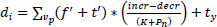	(10)

The denoising of medical images is detected by using Eq. (**10**) from the classification model, in which the variance is improved if noise is present in the image. If there is a lower variance, then the denoising is detected, and it is *d_i_*. In this method, both incremental and decremental learning are addressed based on knowledge of transfer learning. In this methodology, the transfer layers indicate the features and patterns of the medical image. Denoising is pragmatic in this equation and provides better detection of medical images. The prediction is performed in this transfer learning framework, and it is formulated in Eq. (**11**).

**Table:** 

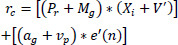	(11)

The prediction is performed in this transfer learning based on Eq. (**11**), where the match of the noise pixel and the variance is mapped with the pre-trained method, and it is represented as *r_c_*. The pre-trained model involves n-number of layers, where diagnostic and convergence denoising are identified. In this category, the training set for the pixel distribution increases, and then the variance decreases, which are inversely proportional to each other. Thus, the prediction measures the matching of the medical image that deploys the noisy pixel in the image. Based on this, the prediction progressed in the pre-trained model. The noise rate of the image is identified from this prediction, and it is evaluated in Eq. (**12**).

**Table:** 

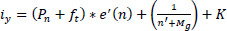	(12)

The noise rate of the image is identified using Eq. (**12**), where the transfer learning process for the n-number of layers is represented as *i_y_*. The noise rate is *n’*, which must be reduced in this work by using transfer learning. This state of processing is approached with knowledge-based training in images. *i_y_* reduction process is presented as a pseudocode in Algorithm **1**.

**Algorithm 1:** *i_y_* Reduction Pseudocode.

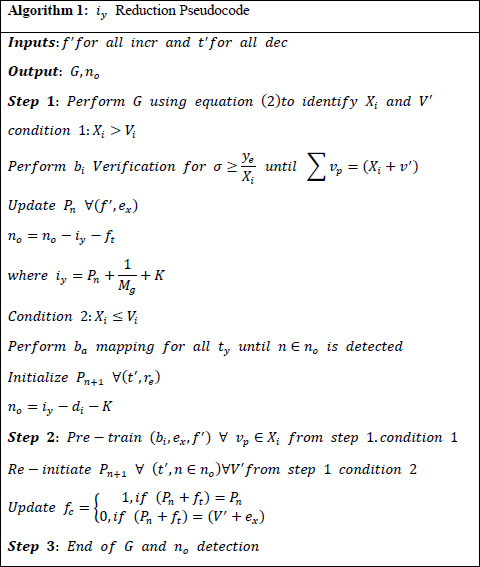

The training phase of the medical image provides an efficient denoising rate where the noisy pixels are identified in the initial layer of the transfer layers. If the noise rate is identified as higher, the training phase is used to reduce this step. From this approach, the knowledge is shared with the next layer. The scope of this paper is to increase the precision of diagnosis, as expressed in Eq. (**13**).

**Table:** 

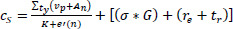	(13)

The precision diagnosis is improved in this proposed work, and it is represented as *c_s_*, in this equation, the correlation factor is used to provide the pixel distribution and variance where the categorization is measured. The re-training model is introduced in this transfer layer, where the training of the medical image is used to differentiate the noise and denoising. Based on this methodology, DCDS and transfer learning are proposed to identify noisy and normal pixels. This oscillation between the states is useful in improving the variance and normal image classification under controlled noise in medical image processing for diagnosis. In Table **4**, the noise reduction rate for *incr* and *decr* levels is evaluated.

*incr* and *decr* based *i_y_* estimation is presented in Table **4** above. This analysis considers incremental and decremental *f*’ and *t*’ for *n_o_* observed classifications. More specifically, *r_e_* augments to (*Pr* + *M_g_*) demands from *e*’(*o*) to *e*’(*n*) under distinct *t_y_*. Therefore, the available *P_n_* is updated to handle either *incr* or *decr* between successive *fc* processes. This in turn, updates *K* using *σ* for the states pursuing *d_i_* operation. If this operation is state, then *r_e_* is expected *δ* satisfy *f_c_* demands to maximize *i_y_.*

A public chest CT scan dataset with Gaussian-like structured noise was used to validate the DCDS framework in this study. This demonstrated the feasibility and baseline performance of our distribution-based compressed denoising scheme; however, we agree that it does not adequately evaluate the method's adaptability to other noise models or imaging modalities like MRI, X-ray, and ultrasound, which have different noise characteristics. Statistical modeling of pixel distributions and correlation patterns is the DCDS approach's main strength, allowing for noise type generalization. We acknowledge that the framework's current implementation lacks a noise-type identification module and a noise-profile-specific adaptive denoising approach.

Our future research will overcome these constraints in the following ways:

#### 2.1.1 Comprehensive Noise-Type Benchmarking

DCDS will be tested in Gaussian, salt-and-pepper, speckle, and Poisson noise settings. This will enable us to evaluate the framework's denoising performance in both single and mixed noise circumstances.

#### 2.1.2 Multi-Modality Validation

To add datasets from other imaging modalities like MRI (which typically has Rician noise), ultrasound (which has speckle noise), and X-rays (which may have low-dose Poisson noise). This will assess the DCDS framework's cross-modal adaptability and resilience.

#### 2.1.3 Adaptive Noise-Aware Enhancements

To investigate pre-denoising noise type estimators and modality-specific tuning layers to dynamically change the model's responsiveness to noise characteristics, boosting generalizability.

The current study serves as a proof of concept, but more research is needed to ensure the DCDS framework works reliably across various noise profiles and imaging modalities. We will address this in future work to increase clinical applicability.

## 3 RESULT ANALYSIS

**Fig. (5):**
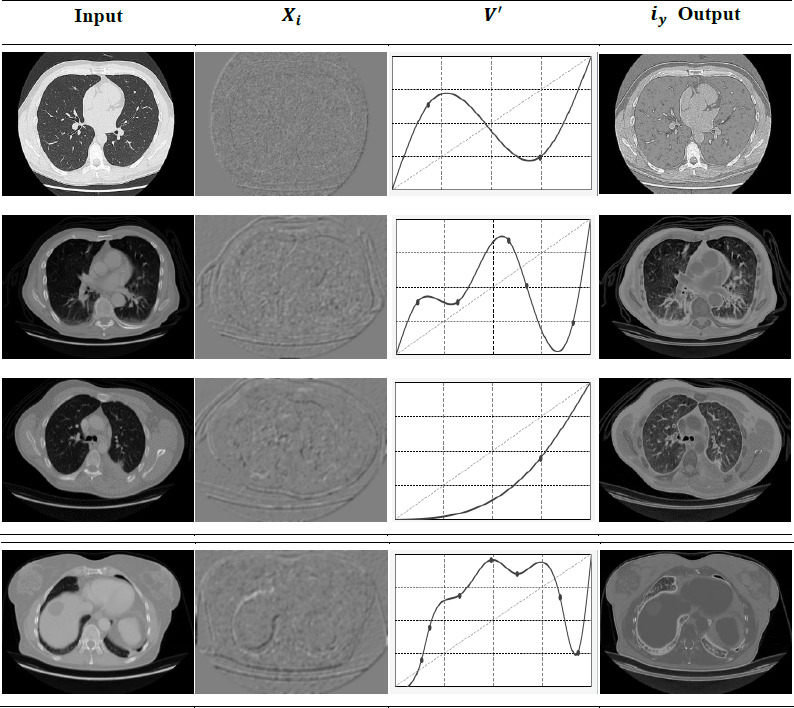
Sample image results.

The results are separated into experimental results and a discussion of comparative analysis. A set of sample image results using experimental analysis is presented in Fig. (**5**). This experiment is conducted using the images given in (Chest CT-Scan images Dataset (kaggle.com)). This dataset provides noisy CT images for detecting lung cancer, which are classified as 70% for training, 20% for testing, and 10% for validation. The distribution, variance, and noise-reduced image illustrations are presented in the following (Fig. **5**).

The comparative analysis discussion is presented using variance detection, noise reduction rate, diagnosis precision, mean error, and analysis time. The variable factors are the number of learning states, ranging from 1 to 12, and the training steps, ranging from 100 to 600. These metrics are studied using the PSNR variants under 4 ranges: 24dB-26dB, 26dB-28 dB, 28dB-30dB, and 30dB-32dB.

### 3.1 Variance Detection

The variance detection for the proposed work increases when the pixel distribution is optimized for denoising medical images. The categorization is involved in this approach for the pixel distribution and variance. Here, the correlation factor is used to deploy the incremental and decremental concept distribution to verify mutual knowledge noise for the transfer between the training and the learning state. This state of computation for variance detection is observed in the categorization process, and it is represented as.


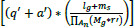


**Fig. (6):**
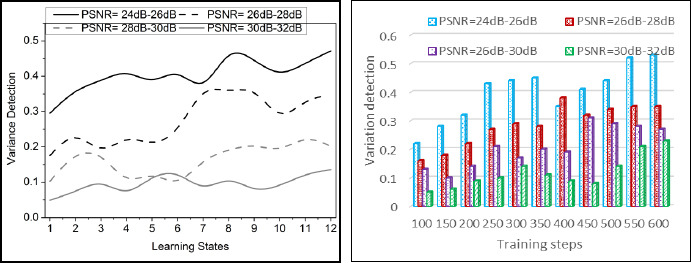
Variance detection analysis.

The acquisition of medical images is used in this case, where the region of detection is performed, and the value and features for the analysis are included. The analysis is carried out to evaluate the value and features of the medical image and provides efficient computation that transfers knowledge to the next layer. This variance detection is processed through transfer learning, where knowledge sharing is detected and shows improved variance. Thus, the detection of categorized variance is enhanced in this work (Fig. **6**).

### 3.2 Noise Reduction Rate

**Fig. (7):**
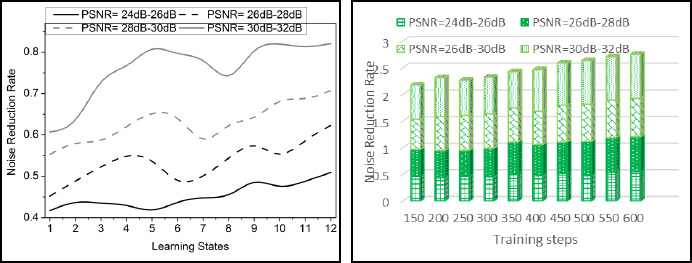
Noise reduction rate analysis.

The noise reduction rate is improved in this paper, including denoising the medical image. Thus, the noise is detected in the initial layer of the transfer learning network, which is then trained and forwarded to the next layer. Forwarding the image to the next layer is associated with the lesser noise. By following this process to the n-number of layers, the processing is carried out to improve noise detection in the layers. The base model acquires the image and processes it to the transfer layers, where the pre-trained model is used in this case, and it is formulated as (*v_p_* * *M_g_*(0) + …*M_g_*(*n*)). In these layers, convergence to optimal denoising is addressed and shows reliable computation. The variance and the pixel distribution are used in this transfer learning. Based on this approach, the transfer layers are interlinked to each other and provide efficient detection of noisy pixels in the image. From the detection process, the pre-trained model is involved in the distribution verification process, where mutual knowledge is shared. Thus, the noise reduction shows better processing of this proposed method (Fig. **7**).

### 3.3 Diagnosis Precision

In Fig. (**8**), diagnosis precision is enhanced by examining the prediction process. In this learning network, the pre-trained model is developed with precision improvement. Here, it states the pre-trained model for pixel distribution and variance. The incremental and decremental processing steps indicate mutual knowledge-sharing and denoising strategies.

The n-number of layers is used to introduce noise-free handling throughout the layer transmission. Eq. (**13**) states the diagnosis of precision improvement, and it is represented as.


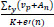


Here, the pre-trained model is used for the analysis phase, which includes extracting regions. Knowledge sharing is performed to improve medical image processing. In this step, both the denoising and the correlation factor are stated. This factor relies on the pixel distribution and variance, which states the density and resolution. The value and features are considered, providing quality in image processing. In this observation, the diagnosis precision is improved for the image based on quality development.

### 3.4 Mean Error

**Fig. (9):**
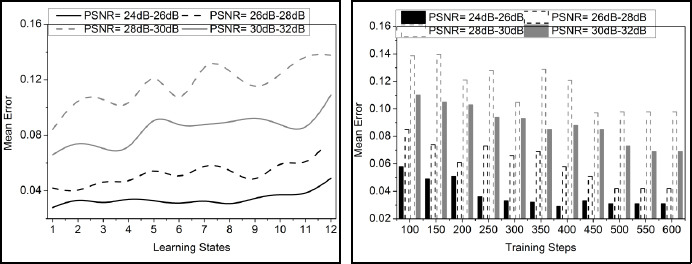
Mean error analysis.

The mean error is lower in the proposed method using a pre-trained model for detection. The analysis is carried out for the pixel distribution and variance, where the knowledge sharing is measured in the n-number of layers. Here, the noisy pixel is detected for mutual knowledge sharing among the pixel distributions and variances. Fine-tuning is introduced in this transfer learning, where the error rate is addressed and reduced. Based on this, the structural processing of layers in this network is associated with the correlation factor. The classification model is proposed for the variance improvement and decrement for the analysis of the noisy region detection, and it is equated as 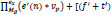

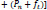
. In this process, the pre-trained model converges to an optimal denoising solution for the medical images. This analysis refers to the quality of the image, which indicates the patterns and features that the image exhibits. This assessment detects the mean error in the preliminary step and reduces it using knowledge-based processing (Fig. **9**).

### 3.5 Analysis Time

In Fig. (**10**), the analysis time for the proposed work decreases, which is associated with detecting noisy pixels in the medical image. This observation level describes the fine-tuning approach, where the pre-trained model involves pixel matching and denotes that it is noisy. If the pixel distribution increases, the variance decreases, which is inversely proportional to each other. The denoising is formulated in Eq. (**10**), where the detection is analyzed for the variance improvement, and it is represented as.


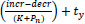


The prediction model is used in this case, where the incremental and decremental processes are involved to better knowledge sharing. Knowledge sharing is processed in this pre-trained model, where it is classified. Here, the prediction using transfer learning indicates knowledge sharing along with the prediction from which the existing model is developed for this medical image detection. Thus, the analysis time is reduced in this approach by performing matching through prediction, which provides efficient processing with transfer learning.

Although the DCDS framework proposed has shown promising performance in experimental settings, it has not yet been subjected to formal testing in actual clinical contexts. Because clinical validation ensures that the model satisfies severe standards related to diagnostic accuracy, visual clarity, and compatibility with real-world clinical procedures, this is an essential limitation that must be taken into consideration. Without this kind of validation, it is difficult to determine whether or not the performance of the framework will translate well in practice. Due to variations in data heterogeneity, workflow restrictions, and user expectations, even research models that perform exceptionally well may not be able to meet the requirements of real-world clinical situations. In the projects we have planned for the future, we intend to collaborate with imaging departments and medical professionals in order to carry out rigorous clinical studies. Specifically, this will entail incorporating the DCDS framework into clinical review pipelines, receiving feedback from specialists regarding the diagnostic usability, and comparing the results to the requirements that have been established in order to guarantee that the approach is safe, trustworthy, and advantageous for clinical deployment. Medical imaging noise reduction and diagnosis accuracy are improved by the DCDS framework. As seen in the design, medical picture pixel distributions and variances are analyzed first. It then uses training images to construct correlations to interpret data trends—incremental or decremental—to guide feature learning. The framework uses pre-trained models to translate this knowledge into noise-filtering categorization. This pipeline is resilient yet computationally intensive. DL and transfer learning, especially with multi-layer network architectures and feature correlations, require significant processing power and extended training times. In time-sensitive clinical applications, pre-training decreases training complexity, although inference can still be difficult. Future work should optimize the DCDS framework for healthcare deployment. Develop lightweight architectures, use model compression methods like pruning and quantization, and investigate efficient inference methodologies to retain performance and reduce computational load. These improvements will match denoising with real-time diagnostic system operations.

## 4 DISCUSSION

The DCDS framework effectively denoises chest CT scan images, as demonstrated by experimental validation using a publicly accessible Kaggle dataset comprising 613 training and 314 testing images. This dataset consists of a typical set of chest CT images for testing our approach, making it sufficient for the current experimental scope. Given the variety in noise characteristics among imaging modalities, equipment types, and patient situations, a single dataset may reduce diversity and representativeness. Using a single dataset may also lead to overfitting, which can impact model generalization in clinical settings. We will expand the dataset base to include MRI, X-ray, and ultrasound to make the DCDS architecture more robust and applicable. This would enable cross-modality validation and ensure the suggested technique works reliably in a broader range of clinical contexts.

The primary method used in this study to evaluate the effectiveness of denoising is PSNR. PSNR is a common approach to measuring the accuracy of a picture, but it does not necessarily match how people see things or how easy it is for doctors to understand. Some denoising methods can get high PSNR values, but they add artefacts or blur that make it harder to understand medical images for diagnosis. The performance analysis is not as complete as it could be because it lacks additional evaluation measures, including the structural similarity index, perceptual quality assessments, or visual grading by specialists.

The DCDS framework introduces a distribution-based compressed denoising scheme. The model learns from statistical measures of pixel variance and correlation patterns across training images and maps these distributions into feature spaces using pre-trained networks. Without a transparent mapping of these modifications, clinical consumers may be wary of the outputs. We aim to utilize explainable AI in future framework versions to address this essential gap. Integrating the following is suggested:

- Saliency Maps show where the model focuses during denoising.
- Attention Mechanisms and Attention Heatmaps display pixel-level relevance scores, highlighting how the model assigns diagnostic weights to essential locations.

To determine how input features (pixels or areas) affect the model's denoising output, utilize feature attribution techniques, such as SHAP, LIME, and Integrated Gradients. Clinicians can assess if essential anatomical or pathological features are preserved or altered during noise removal using these explainable AI methods, boosting confidence in the model's usability and safety. These visual explanations can also be used as an audit trail to verify denoising quality and diagnostic interpretation.

## CONCLUSION

This article introduces a DCDS to reduce noisy pixels in medical images to support diagnosis precision. The proposed scheme is aided by transfer learning to define and validate incremental and decremented distribution. First, the distribution, type, and variance are segregated from the input image. The correlation between actual and noisy pixels is performed using a set of training images. The learning process ensures pre-trained and training conditions based on incremental and decremented functions that resume compression of noisy pixels through state changes. The state changes are defined for high correlation and high variance, which are updated based on the pre-trained layers. Mapping different layers between the actual and training inputs detects the precise noise rate and variance detection sequentially. Therefore, the PSNR ranges are high at any pre-trained state with high correlation, thereby reducing the noise. The noise-reduced pixel distributions are combined to form an incremental image for better diagnosis. The suggested denoising approach may produce less-than-ideal results when applied to small or biased datasets, as its effectiveness depends on the size and quality of the training set. To ensure interoperability with various imaging equipment and diagnostic workflows, further research should investigate optimization ways to reduce computational complexity and enable the denoising scheme to be implemented in real-time in clinical situations.

## Figures and Tables

**Fig. (1):**
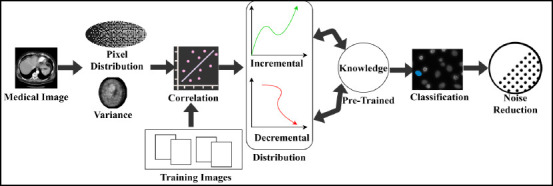
Schematic illustration of the DCDS scheme.

**Fig. (8):**
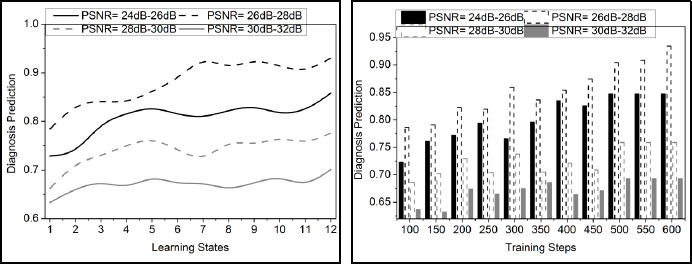
Diagnosis precision analysis.

**Fig. (10):**
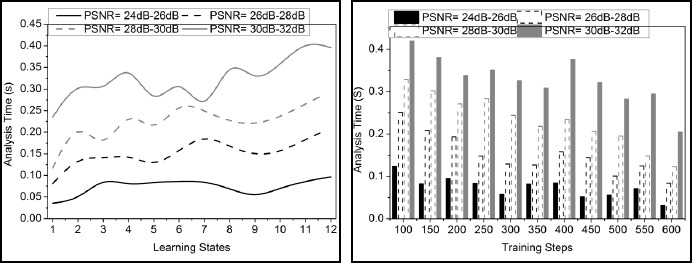
Analysis time.

**Table 1:** *X_i_* and *V’* for different *G.*

***r’***	***q*’ + *a*’**	***y_e_+ u_s_***	***X’***	***V*’**
2	-	*M_g_*/*n*_0_	0.61	0.18
	1/*l_g_*	-	0.87	0.07
	1/*l_g_*	*M_g_*/*n*_0_	1	0
4	-	*M_g_*/*n*_0_	0.66	0.24
	1/*l_g_*	-	0.89	0.1
	1/*l_g_*	*M_g_*/*n*_0_	0.94	0.05
6	-	*M_g_*/*n*_0_	0.55	0.36
	1/*l_g_*	-	0.73	0.19
	1/*l_g_*	*M_g_*/*n*_0_	0.85	0.09
8	-	*M_g_*/*n*_0_	0.51	0.41
	1/*l_g_*	-	0.68	0.28
	1/*l_g_*	*M_g_*/*n*_0_	0.81	0.12

**Table 2:** ***b_i_*** categorization for *r*’.

***r'***	***X_i_* + *V*’**	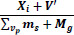	***σ***	***b_i_***	**Type**
2	True	False	0.72, 0.65, 0.84	1.105	*incr*
	False	True	0.72, 0.69, 0.73	1.07	*incr*
	True	True	0.85, 0.72, 0.65	1.19	*incr*
4	True	False	0.92, 0.69, 0.81	0.58	*decr*
	False	True	0.74, 0.72, 0.65	0.5275	*decr*
	True	True	0.65, 0.65, 0.84	0.535	*decr*
6	True	False	0.85, 0.81, 0.72	0.395	*decr*
	False	True	0.81, 0.72, 0.65	0.3633	*decr*
	True	True	0.78, 0.98, 0.81	0.429	*decr*
8	True	False	0.65, 0.65, 0.84	0.268	*decr*
	False	True	0.87, 0.98, 0.81	0.3325	*decr*
	True	True	0.98, 0.81, 0.72	0.3137	*decr*

**Table 3:** ***r_e_*** for different pre-training values.

***vp***	***e_x_***	***b_i_***	***r*’**	***t_y_***	***r_e_***	***b_i_* Computed as**
0	0	0	1	9	0.35	*σ + a_g_*
0	0	1	0	5	0.32	*σ + a_g_*
0	1	0	0	8	0.28	*X_i +_ V’*
1	0	0	0	2	0.45	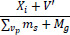
0	0	1	1	10	0.21	*X_i +_ V’*
0	1	1	0	8	0.35	*σ + a_g_*
1	0	0	0	1	0.5	(*σ + a_g_*) *+* (*t_r_* * *M_g_*)
1	0	1	1	7	0.4	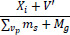
0	1	1	1	8	0.35	*σ + a_g_*
1	1	1	0	10	0.23	*X_i +_ V’*
1	0	1	1	11	0.2	*X_i +_ V’*
1	1	0	1	10	0.22	*X_i +_ V’*
1	1	1	1	11	0.2	*X_i +_ V’*

**Table 4:** Noise reduction rate for *incr* and *decr*.

***incr***	***decr***	***b_i_***	***fc* (%)**	***i_y_***
2	-	0.78	38.14	0.73
4		0.69	74.25	0.49
6		0.84	73.52	0.54
8		0.94	48.51	0.73
-	8	0.61	52.1	0.58
	6	0.68	81.6	0.65
	4	0.67	75.6	0.74
	2	0.71	74.25	0.79
Max = 8	Min = 2	0.84	82.21	0.83
Min = 2	Max = 8	0.74	71.2	0.61
Equal = 4	Equal = 4	0.81	80.25	0.72
Equal = 6	Equal = 6	0.65	79.32	0.65

## Data Availability

The data used to implement the proposed method is available in the public domain at the following web link: https://www.kaggle.com/datasets/mohamedhanyyy/chest-ctscan-images

## LIST OF ABBREVIATIONS

AI: Artificial Intelligence
CNN: Convolutional Neural Network
CT: Computed Tomography
DCDS: Distribution-based Compressed Denoising Scheme
DL: Deep Learning
LDCT: Low-Dose Computed Tomography
MRI: Magnetic Resonance Imaging
PAM: Photoacoustic Microscopy
PSNR: Peak Signal-to-Noise Ratio
